# Second-Generation Parenteral Antipsychotic (Olanzapine) as a First-Line Treatment for Acute Undifferentiated Agitation in the Emergency Department in Comparison With Haloperidol

**DOI:** 10.7759/cureus.40226

**Published:** 2023-06-10

**Authors:** Adheera P Singh, N. T Murali Mohan

**Affiliations:** 1 Emergency Medicine, Vydehi Institute of Medical Sciences and Research Centre, Bangalore, IND

**Keywords:** agitated delirium, alcohol/drug intoxication, traumatic brain injury, sedation, antipsychotic drugs, emergency department, haloperidol, olanzapine, acute undifferentiated agitation

## Abstract

Background

Acutely agitated patients are common in the emergency department (ED). Given the myriad aetiologies of the clinical conditions that can produce agitation, such a high prevalence is unsurprising. Agitation is a symptomatic presentation, not a diagnosis, secondary to a psychiatric, medical, traumatic, or toxicological condition. Most literature on the emergency management of agitated patients is from psychiatric populations, not generalised to EDs. Benzodiazepines, antipsychotics, and ketamine have been used to treat acute agitation. However, a clear consensus is lacking.

Objectives

The objectives are to study the effectiveness of intramuscular (IM) olanzapine as a first-line treatment for rapid tranquillisation in undifferentiated acute agitation in the ED and compare the effectiveness of sedatives to control agitation in etiologically divided groups per the following preassigned protocols: Group A: Alcohol/drug intoxication (olanzapine vs haloperidol), Group B: Traumatic brain injury (TBI) with or without alcohol intoxication (olanzapine vs haloperidol), Group C: Psychiatric conditions (olanzapine vs haloperidol and lorazepam), and Group D: Agitated delirium, organic causes (olanzapine vs haloperidol).

Methods

This 18-month prospective study included acutely agitated ED patients between 18 and 65.

Results

A total of 87 patients between 19 and 65 were included, all with a Richmond Agitation Sedation Scale (RASS) score between +2 and +4 at presentation. Nineteen of the 87 patients were managed as acute undifferentiated agitation, and 68 were assigned to one of the four groups. In acute undifferentiated agitation, IM olanzapine 10 mg effectively sedated 15 (78.9%) patients within 20 minutes, whereas the remaining four (21.1%) were sedated with a repeat IM olanzapine 10 mg over the next 25 minutes. In 13 patients with agitation due to alcohol intoxication, zero out of three with olanzapine and four out of 10 (40%) with IM haloperidol 5 mg were sedated within 20 minutes. In patients with TBI, two out of eight (25%) receiving olanzapine and four out of nine (44.4%) receiving haloperidol were sedated within 20 minutes. In acute agitation secondary to psychiatric disease, olanzapine sedated nine out of 10 (90%), and haloperidol with lorazepam sedated 16 out of 17 (94.1%) within 20 minutes. In patients with agitation secondary to organic medical conditions, olanzapine rapidly sedated 19 out of 24 (79.1%), whereas haloperidol sedated one out of four (25%).

Interpretation and conclusion

IM olanzapine 10 mg is effective for rapid sedation in acute undifferentiated agitation. Olanzapine is superior to haloperidol in agitation secondary to organic medical conditions and is as efficacious as haloperidol with lorazepam in agitation due to psychiatric diseases. However, in agitation due to alcohol intoxication and TBI, haloperidol 5 mg is slightly better, although not statistically significant. Olanzapine and haloperidol were well tolerated by Indian patients in the current study, with minimal side effects.

## Introduction

Acutely agitated patients are not an uncommon presentation to the emergency department (ED). This is not surprising, since agitation can be the only or one of the symptoms in a variety of clinical conditions. Patients with traumatic brain injury (TBI), alcoholic or drug intoxication, neurological conditions, certain medical or metabolic disorders, and various psychiatric conditions can present with acute agitation as the primary symptom. A few Western studies have reported a prevalence rate between 2% and 10% of all emergency admissions [1].

Acutely agitated patients in the ED can cause harm and/or injury to ED personnel, attending physicians, nurses, and sometimes even to themselves. This can jeopardise their immediate treatment and eventual recovery. Not infrequently, agitation escalates to violence in the ED, which is disruptive and potentially carries a greater risk of injury to ED personnel [2]. According to Western literature, close to 40% of ED personnel have experienced violence at some point in their career [3]. 

Most literature on the emergency management of acutely agitated patients comes from psychiatric EDs and, hence, mainly pertains to psychiatric conditions. Only recently studies and trials on the management of agitated patients have been reported from emergency departments in general hospitals. Different drug classes including benzodiazepines, first-generation antipsychotics, second-generation antipsychotics, and, more recently, ketamine have been used to manage acutely agitated patients. However, a general consensus on the use of a particular drug class in a certain condition is lacking. Some United States and Canadian guidelines recommend lorazepam, haloperidol, olanzapine, or midazolam, singly or in combination [4]. In the United Kingdom, the National Institute for Health and Care Excellence (NICE) guidelines recommend lorazepam as the first choice [5], and Australian guidelines recommend droperidol [6] as the first-line management in acute agitation. Clearly, a consensus is lacking in the choice of drug for the management of acute agitation resulting from different etiologies. Recommendations differ not only in different countries but also among hospitals in the same country or region. In India, reports addressing the problem are sparser or even nonexistent.

Acutely agitated patients in the ED must be promptly controlled with verbal de-escalation, physical restraints (when absolutely necessary), or pharmacological intervention, whichever helps best. Concurrently, assessment of possible etiology must begin and prompt treatment executed. However, the challenge in the ED is choosing a drug that is most effective, with rapid onset and minimal risks and side effects. 

## Materials and methods

This was an 18-month observational, cohort study comprising 87 agitated patients presenting to the ED of the Vydehi Institute of Medical Sciences and Research Centre, Bangalore, India between January 19, 2019, and June 20, 2020. The inclusion criteria were: patients aged 18 to 65 years, with a relative willing to give informed consent, with a diagnosis as per the Diagnostic and Statistical Manual of Mental Disorders (DSM-5), which defines agitation as ‘excessive motor activity with a feeling of inner tension.’ The severity was measured as per the Richmond Agitation Sedation Scale (RASS). All patients with scores between +2 and +4 were included in the study. The exclusion criteria were: a relative not willing to give informed consent, pregnant women, and paediatric patients.

Methodology

All acutely agitated patients between the ages of 18 and 65 years presenting to the ED were assessed by the RASS. All patients with RASS scores between +2 and +4 with an attendant agreeing to sign a contract were included in the study. In agitated patients where verbal de-escalation was impractical or impossible, a four-point restraint was applied by the ED staff.

Patients with known aetiology at onset (e.g. history of alcohol intoxication, head injury, psychiatric illness, or known medical cause of agitated delirium) were assigned to the respective group and treated as per preassigned protocols. All patients with acute undifferentiated agitation were treated with an intramuscular (IM) injection of olanzapine 10 mg. A repeated second dose was given if adequate sedation was not achieved within 20 minutes. After the initial sedating dose, an assessment for adequate sedation was made at 20 minutes, noted by achieving a RASS score between 0 and -2.

In patients not sedated within 20 minutes of the first injection, a repeat injection of the same drug in the same dose was administered. Assessment for sedation was performed at 15, 20, and 25 minutes after the second injection, targeting a RASS score between 0 and -2. There was continued monitoring at half-hourly intervals afterwards to note any recrudescence of agitation assessed by a return to a RASS score between +2 and +4 and the time noted from the initial sedation for that event.

Once the patient's agitation was controlled, further assessment and monitoring were conducted in a stepwise fashion: obtaining history details, a quick clinical exam (general and systemic), and initiating vital sign monitoring of the pulse, blood pressure, respiration rate, and oxygen saturation. In addition, venous access was obtained and urgent routine laboratory blood tests were conducted including that for blood sugar, kidney function, liver function, electrolytes, and other tests indicated by a suspected medical condition, such as a toxicology screen. In addition, an ECG/echocardiogram (Echo), if indicated, and a computed tomography (CT) head exam was done for suspected trauma/stroke.

After assessment with the history/examination and laboratory tests, a provisional diagnosis was made, and the patients were divided into the following four groups: Group A for alcoholic intoxication, Group B for TBI with or without alcohol intoxication, Group C for psychiatric conditions, and Group D for agitated delirium due to medical conditions. The initial sedation or additional sedation for differentiated patients was also categorised as per the group stratification. Group A was randomised into two groups, Group A1 with IM olanzapine 10 mg and Group A2 with IM haloperidol 5 mg. Group B was randomised into Group B1 with IM olanzapine 10 mg, and Group B2 with IM haloperidol 5 mg. Group C was randomised into Group C1 with IM olanzapine 10 mg and Group C2 with IM haloperidol 5 mg and lorazepam 2 mg. Group D was randomised into Group D1 with IM olanzapine 10 mg and Group D2 with IM haloperidol 5 mg. 

Outcome measures

The control or effective treatment was measured by achieving a RASS score between 0 and -2 in the treated patient. In addition, the time taken to achieve sedation and the need for a second sedating/tranquillising drug, if required, and the duration to achieve sedation after the second dose were assessed.

Data entry and statistical analysis

The collected data were transformed into variables, coded, and entered in Microsoft Excel (Microsoft Corporation, Redmond, Washington, United States). The data were analysed and statistically evaluated using IBM SPSS Statistics for Windows, Version 21.0 (Released 2012; IBM Corp., Armonk, New York, United States). The quantitative data were expressed as the mean±SD or median with an interquartile range (IQR). In contrast, the qualitative data were expressed in percentages, and the statistical differences between the proportions were tested using the chi-square test or Fisher’s exact test. A p-value of less than 0.05 was considered statistically significant.

## Results

A total of 87 acutely agitated patients aged 18 and 65 years, presenting to the ER during the study period, were included in the study. Most (n=36; 41.4%) were between 26 and 35 years, and a large number (n=70; 80.5%) were males. The demographic profile of the study subjects is presented in Table 1.

**Table 1:** Demographic profile of study subjects

Characteristics	Number of patients		%
Age group			
18-25 years		19	21.8
26-35 years		36	41.4
36-45 years		16	18.4
46-55 years		7	8.0
56-65 years		9	10.3
Gender			
Male	70		80.5
Female	17		19.5

All 87 patients had RASS scores between +2 and +4 at presentation to the ER. Most of these patients, 52 (59.8%), had a score of +2, and only one subject had a score of +4. Out of these 87 patients, 19 had undifferentiated agitation on admission, whereas 68 patients could be assigned to one of the four groups at the initial presentation. Of these 68 patients, 13 patients were assigned to Group A (alcohol intoxication), 17 patients to Group B (TBI with or without alcohol intoxication), 19 patients to Group C (known psychiatric disorder) and 19 patients to Group D (agitated delirium due to known organic causes) (Table 2).

**Table 2:** Aetiology of agitation

Diagnosis	Initial diagnosis	Final diagnosis
Alcohol intoxication	13	13
Traumatic brain injury with or without alcohol intoxication	17	17
Psychiatric conditions	19	27
Agitated delirium–organic causes	19	28
Undiagnosed	19	2

All 19 patients classified as having undifferentiated agitation with no known cause at presentation were given an IM injection of olanzapine 10 mg. None of these 19 patients responded to verbal de-escalation, and 12 had to be physically restrained to receive the IM injection. Of the 19 patients receiving IM olanzapine 10 mg, 15 achieved adequate sedation at 20 minutes (78.9%), revealing that IM olanzapine 10 mg was very effective in achieving rapid tranquillisation in this group (Table 3). The remaining four patients were given an additional 10 mg of olanzapine after 20 min of the first dose. All four patients achieved adequate sedation within the next 25 minutes. Table 4 presents the relative frequency of the final RASS score achieved in this group of patients.

**Table 3:** Sedation achieved in 20 minutes in the undifferentiated group (n=19)

Sedation achieved	No.	%
Yes	15	78.9
No	4	21.1

**Table 4:** RASS score at sedation in the undefined group (n=19) RASS: Richmond Agitation Sedation Scale

RASS Score at sedation	No.	%
0	13	68.4
-1	4	21.0
-2	2	10.5

After further clinical evaluation and investigation, the 19 patients were assigned to one of the four study groups, along with the other patients already put in their respective groups at the initial presentation. The final frequency distribution is listed in Table 2. Two patients with undifferentiated agitation could not be assigned to any particular group in the final analysis because they left the ER before the completion of all investigations.

Group A (alcohol intoxication)

Thirteen patients were assigned to Group A (alcohol intoxication). Of these, three received injections of olanzapine 10 mg, and 10 patients received IM haloperidol 5 mg (Table 5).

**Table 5:** Drugs given to study subjects in Group A (n=13)

Initial drugs given	No.	%
Haloperidol	10	76.9
Olanzapine	3	23.1

None of the three patients receiving IM olanzapine 10 mg was sedated at 20 minutes. After receiving a repeat IM olanzapine 10 mg, one patient was sedated after 15 minutes and two were sedated 20 minutes after the repeat injection. Ten patients in this group were given IM haloperidol 5 mg. Four of these patients achieved sedation at 20 min. The remaining six patients were given repeat IM injections of haloperidol 5 mg. Of these, six patients were given repeat injections, five were sedated after 15 minutes and one after 25 minutes. Table 6 presents the duration to achieve sedation after a repeat injection.

**Table 6:** Duration until sedation after repeated sedation dose in Group A

Duration until sedation	Haloperidol (n=6)	Olanzapine (n=3)
35 min	5	1
40 min	0	2
45 min	1	0

The comparison of the injection of olanzapine 10 mg and haloperidol 5 mg in achieving adequate sedation after the first dose of respective injections was not statistically significant (p = 0.49) (Table 7), and no inferences could be derived because of the small number of subjects in this group. After a second injection of the same sedating agent (Table 8), both drugs were identical in achieving the requisite sedation within 45 minutes of the injection. No side effects were noted with either of these drugs in this group. 

**Table 7:** Sedation achieved at 20 minutes with different drugs in Group A (n=13)

Sedation achieved at 20 min	Haloperidol (n=10)	Olanzapine (n=3)	P value
No	6 (60.0%)	3 (100.0%)	0.49
Yes	4 (40.0%)	0	

**Table 8:** Repeat drugs given to study subjects who did not achieve sedation in Group A (n=9)

Type of drugs given	No.	%
Haloperidol	6	66.7
Olanzapine	3	33.3

Group B (TBI with or without alcohol intoxication)

Seventeen patients were in Group B, of which eight received IM olanzapine 10 mg, and nine received IM haloperidol 5 mg (Table 9). Of the eight patients receiving an injection of olanzapine, only two were adequately sedated at 20 minutes (Table 10). Of the remaining six patients receiving repeated IM injections of 10 mg of olanzapine, three were sedated at 15 minutes, one at 20 minutes, and two at 25 minutes after the repeat injection (Table 11).

**Table 9:** Drugs given to study subjects in Group B (n=17)

Initial Drugs given	No.	%
Haloperidol	9	52.9
Olanzapine	8	47.1

**Table 10:** Sedation achieved at 20 minutes with the assessed drugs in Group B (n=17)

Sedation achieved at 20 min	Haloperidol (n=9)	Olanzapine (n=8)	P value
No	5 (55.6%)	6 (66.7%)	0.62
Yes	4 (44.4%)	2 (33.3%)	

**Table 11:** Duration until sedation in Group B

Duration until sedation	Haloperidol (n=9)	Olanzapine (n=8)
20 min	4 (44.4%)	2 (25.0%)
35 min	2 (22.2%)	3 (37.5%)
40 min	2 (22.2%)	1 (12.5%)
45 min	1 (11.1%)	2 (25.0%)

On comparison of the IM olanzapine 10 mg and haloperidol 5 mg on achieving adequate sedation after the first injection, no statistical difference was observed between the injection of haloperidol (44.4%) and olanzapine (33.3%) with a p-value of 0.62. However, after the second injection of the respective sedating agents, both were equally efficacious (100%) in achieving adequate sedation within 45 min of the first injection. In this group, one patient developed hypotension, which responded to conservative treatment. No respiratory or CNS side effects were noted with either drug (Table 12).

**Table 12:** Side effects of the assessed drugs in Group B (n=17)

Side effects	Haloperidol (n=9)	Olanzapine (n=8)
Cardiovascular: hypotension	1 (11.1%)	1 (12.5%)
Respiratory depression	0	0
Extrapyramidal symptoms	0	0

Group C (psychiatric conditions)

Group C comprised 27 patients. In this group, 10 patients (37%) were given IM olanzapine 10 mg and 17 patients (63%) were given IM haloperidol 5 mg plus IM lorazepam 2 mg. The frequency distribution of the tranquillising agent is indicated in Table 13.

**Table 13:** Drugs given to study subjects in Group C (n=27)

Initial Drugs given	No.	%
Haloperidol with Lorazepam	17	63.0
Olanzapine	10	37.0

Of the 10 patients receiving an IM olanzapine 10 mg, nine (90%) were adequately sedated at 20 minutes. Of the 17 patients receiving haloperidol and lorazepam 2 mg, 16 (94.1%) were adequately sedated at 20 minutes (Table 14). In this group, injections of olanzapine (90%) and haloperidol with lorazepam (94.1%) were equally efficacious in achieving adequate sedation at 20 minutes after the first injection, which was statistically significant (p = 1). One patient in each group received a second dose of the sedating agent 20 minutes after the initial dose. One of these two patients received an injection of olanzapine and achieved adequate sedation after 15 minutes, and the other patient received an injection of haloperidol plus lorazepam and achieved sedation after 20 minutes of the repeat injection. After the second injection, both drugs achieved adequate sedation in the remaining patients within 20 minutes of the repeat injection (Table 15).

**Table 14:** Sedation at 20 min with the assessed drugs in Group C (n=27)

Sedation at 20 min	Haloperidol with Lorazepam (n=17)	Olanzapine (n=10)	P value
No	1 (5.9%)	1 (10.0%)	1.0
Yes	16 (94.1%)	9 (90.0%)	

**Table 15:** Duration until sedation in Group C

Duration until sedation	Haloperidol with Lorazepam (n=17)	Olanzapine (n=10)
20 min	11 (64.7%)	9 (90.0%)
25 min	5 (29.4%)	0
35 min	0	1 (10.0%)
40 min	1 (5.9%)	0

In this group, only one patient developed significant side effects. One patient receiving an injection of haloperidol with lorazepam developed dystonia, which settled with IM benztropine mesylate 1 mg. No other cardiovascular (CVS) or respiratory side effects were noted with either drug (Table 16).

**Table 16:** Side effects of the assessed drugs in Group C

Side effects	Haloperidol with Lorazepam (n=17)	Olanzapine (n=10)
Cardiovascular: hypotension	0	0
Respiratory depression	0	0
Dystonia	1 (5.9%)	0

Group D (agitated delirium from organic causes)

Group D comprised 28 patients. Of these, 24 patients (85.7%) received IM olanzapine 10 mg and four (14.2%) received IM haloperidol 5 mg (Table 17). Nineteen of the 24 patients receiving injections of olanzapine and one of the four patients receiving haloperidol achieved adequate sedation after 20 minutes (Table 18).

**Table 17:** Drugs given to study subjects in Group D (n=28)

Initial Drugs given	No.	%
Haloperidol	4	14.3
Olanzapine	24	85.7

**Table 18:** Sedation at 20 minutes in different drugs in Group D (n=28)

Sedation at 20 min	Haloperidol (n=4)	Olanzapine (n=24)	P value
No	3 (75.0%)	5 (20.8%)	0.058
Yes	1 (25.0%)	19 (79.2%)	

Of the five patients given a repeat IM olanzapine 10 mg, three achieved adequate sedation after 15 minutes, one after 20 minutes, and one after 25 minutes. Of the three patients receiving a repeat IM haloperidol 5 mg, two achieved adequate sedation after 15 minutes and one after 25 minutes (Table 19). In this group, when comparing adequate sedation at 20 minutes, IM olanzapine was statistically superior to IM haloperidol 5 mg (p = 0.003). After the repeat injection, adequate sedation was achieved within 45 minutes of the first injection with both drugs.

**Table 19:** Duration until sedation in Group D

Duration until sedation	Haloperidol (n=4)	Olanzapine (n=24)
20 min	1 (25.0%)	19 (79.16%)
35 min	2 (50.0%)	3 (12.5%)
40 min	0	1 (4.16%)
45 min	1 (25.0%)	1 (4.16%)

The only minor side effect noted in this group was tachycardia in one patient and hypoxia in another (settled with minimal oxygen support). Both these minor side effects were noted in patients receiving injections of olanzapine (Table 20).

**Table 20:** Side effects of the assessed drugs in Group D

Side effects	Haloperidol (n=4)	Olanzapine (n=24)
Cardiovascular: Palpitations	0	1 (4.2%)
Hypoxia	0	1 (4.2%)
Extrapyramidal side effect	0	0

Analysis of data irrespective of etiological groups

In the study, 23 (26.4%) out of 87 patients received injections of haloperidol, 17 (19.5%) received injections of haloperidol with lorazepam, and 47 (54%) received injections of olanzapine (Table 21).

**Table 21:** Drugs given to study subjects (n=87)

Type of drugs given	No.	%
Haloperidol	23	26.4
Haloperidol with Lorazepam	17	19.5
Olanzapine	47	54.0

Time Needed to Achieve Adequate Sedation After the First Dose of the Sedating Agent 

After the first injection of the sedating drug, adequate sedation was achieved within 20 minutes in nine of the 23 patients given an injection of haloperidol, 16 of the 17 patients given haloperidol with lorazepam, and 32 of the 47 patients given an injection of olanzapine. The sedation achieved within 20 minutes with a combined injection of haloperidol with lorazepam was statistically highly significant (p = 0.001) compared with the individual injections of haloperidol and olanzapine (Table 22). All patients receiving injections of haloperidol with lorazepam belonged to Group C (psychiatric patients).

**Table 22:** Sedation at 20 minutes in the assessed drugs

Sedation at 20 min	Haloperidol (n=23)	Haloperidol with Lorazepam (n=17)	Olanzapine (n=47)
No	14 (60.9%)	1 (5.9%)	15 (31.9%)
Yes	9 (39.1%)	16 (94.1%)	32 (68.1%)

Time Needed to Achieve Adequate Sedation After the Second Dose of the Sedating Agent 

Of the 14 patients receiving haloperidol injections, nine were sedated after 15 minutes, two after 20 minutes, and three after 25 minutes. In the only patient receiving an injection of haloperidol with lorazepam, sedation was achieved after 20 minutes. Of the 15 patients receiving an injection of olanzapine, seven achieved adequate sedation after 15 minutes, five after 20 minutes, and three after 20 minutes. This statistical comparison was insignificant (p = 0.37) because all three drugs achieved adequate sedation within 25 minutes (Table 23).

**Table 23:** Duration until sedation after second dose of sedating agent

Duration until sedation	Haloperidol (n=14)	Haloperidol with Lorazepam (n=1)	Olanzapine (n=15)
35 min	9 (64.3%)	0	7 (46.7%)
40 min	2 (14.3%)	1 (100.0%)	5 (33.3%)
45 min	3 (21.4%)	0	3 (20.0%)

Time Needed to Achieve Adequate Sedation and Side Effects in General with the Three Drugs or Drug Combinations

In 23 patients receiving an injection of haloperidol, nine (39.1%) were sedated after 20 minutes, nine (39.1%) after 35 minutes, two (8.7%) after 40 minutes, and three (13.0%) after 45 minutes. In 17 patients receiving an injection of haloperidol with lorazepam, 16 (94.1%) were sedated after 20 minutes and one (5.9%) after 40 minutes. In 47 patients receiving an injection of olanzapine, 32 (68.0%) were sedated after 20 minutes, seven (14.9%) after 35 minutes, five (10.6%) after 40 minutes, and three (6.4%) after 45 minutes (Table 24). Thus, examining all drugs or drug combinations together revealed that 57 of the 87 patients (65.5.%) achieved adequate sedation at 20 minutes after a single injection.

**Table 24:** Total time until sedation

Duration until sedation	Haloperidol (n=23)	Haloperidol with Lorazepam (n=17)	Olanzapine (n=47)
20 min	9 (39.1%)	16 (94.1%)	32 (68.0%)
35 min	9 (39.1%)	0	7 (14.9%)
40 min	2 (8.7%)	1 (5.9%)	5 (10.6%)
45 min	3 (13.0%)	0	3 (6.4%)

No significant side effects were observed with any drugs or drug combinations in this study. In the 23 patients receiving injections of haloperidol, only one patient developed hypotension. In the 17 patients receiving injections of haloperidol with lorazepam, only one developed dystonia, and none had CVS or respiratory side effects. In the 47 patients receiving injections of olanzapine, one developed hypotension, one developed tachycardia, one developed mild hypoxia, and none had CNS side effects (Table 25).

**Table 25:** Side effects of the assessed drugs CVS: cardiovascular

Side effects	Haloperidol (n=23)	Haloperidol with Lorazepam (n=17)	Olanzapine (n=47)
CVS: hypotension	1 (4.3%)	0	1 (2.1%)
CVS: palpitation	0	0	1 (2.1%)
Hypoxia	0	0	1 (2.1%)
Dystonia	0	1 (5.9%)	0

## Discussion

IM olanzapine and other atypical antipsychotics have been used to manage acute agitation in psychiatric emergencies for some time. Olanzapine, along with other agents, such as midazolam, ketamine, haloperidol, or droperidol, are used to manage undifferentiated agitation in the ED in different parts of the world. Parenteral olanzapine has become the preferred drug for the initial management of undifferentiated agitation, despite the lack of definitive supportive evidence. This study is the first to evaluate the use of parenteral olanzapine in Indian patients in a general multispecialty hospital emergency setting.

In this study, when IM olanzapine 10 mg was given to undifferentiated acutely agitated patients in the ER, 78.9% of patients were sedated within 20 minutes. Moreover, the remaining 21.1%, upon receiving the second dose 20 minutes after the first injection, were sedated over the next 25 minutes (i.e. within 45 min of the first injection). The results of achieving rapid sedation with IM olanzapine are similar to the only other study of Indian patients by Raveendran et al. [7], which was conducted with psychiatric patients. In that study, adequate sedation was achieved in 87% of the patients within 15 minutes of receiving IM olanzapine 10 mg [7]. The results of this study in achieving rapid and safe tranquillisation with IM olanzapine in acute agitation are similar to other reports in the literature worldwide, including the 2019 meta-analysis by Bak et al. [8-11].

It is generally accepted that Indian patients require lower sedative doses of most sedatives than the Western population [12,13]. Hence, there was an initial apprehension that there would be greater sedative and respiratory side effects with doses similar to those employed in Western studies [13]. However, in this study, like the study on Indian patients conducted by Raveendran et al. [7], no patient developed any adverse effects after 10 mg or even two doses of 10 mg IM olanzapine (given 20 minutes apart). As this study was restricted to adult patients with an upper age limit of 65 years, it is possible that elderly patients may require smaller doses, as reported earlier by Duong et al. [14], among others.

Oversedation has been reported in 3-31% of patients [8,15], movement disorders in 0-5% [7,8,15], CVS adverse effects, including QTc prolongation in up to 3% [11], and hypotension in 0.01-0.02% [16]. However, no patient in this study developed the above side effects. Mild adverse effects, not requiring any intervention, were noted in about 2% of patients.

There is no ‘best’ or ‘ideal’ sedating agent for all categories of patients with acute agitation. Thus, one objective of this study was also to determine the effectiveness of IM olanzapine compared to IM haloperidol or IM haloperidol with lorazepam in four etiological categories: alcohol intoxication, TBI with or without alcohol intoxication, psychiatric diseases, and other medical conditions presenting with acute agitation.

In this study, in patients with acute agitation secondary to alcohol intoxication, IM haloperidol 5 mg was more effective (4/10 patients) compared to IM olanzapine 10 mg (0/3 patients) in achieving rapid tranquillisation within 20 minutes of the injection. One possible reason for the low percentage of sedation with a particular drug dose could be the relative tolerance to the sedating effect in patients with alcohol/drug abuse. However, after repeat injections of the original sedating drugs, sedation was achieved in 100% of patients with both drugs. No significant conclusion could be drawn given the small sample size in this study. Haloperidol has generally been reported to have more efficacy in patients with agitation due to alcohol intoxication in various studies [4,17,18]. In contrast, in another large study by Klein et al., where the majority of patients (88%) had alcohol intoxication, the most effective tranquillising agent to achieve rapid sedation was IM midazolam, while olanzapine 10 mg was more effective than IM haloperidol 5 mg [19].

The choice of pharmacological agents to control acute agitation in patients with TBI remains unclear and unproven. Olanzapine has been reported to reduce agitation in patients with TBI in certain open studies and case series [20-22]. In this study, in patients presenting with acute agitation secondary to TBI (with or without alcohol intoxication), haloperidol 5 mg was slightly more efficacious than olanzapine 10 mg in achieving rapid sedation (44.4% vs 33.3%, respectively), although it was not statistically significant. However, after the second injection of the respective sedating agent, sedation was achieved in 100% of the patients with both drugs within 45 minutes of the first injection. In this study, no other significant side effects were observed except for a single patient each developing hypotension with either drug. Concerns about lowering the seizure threshold and the rare occurrence of a malignant neuroleptic syndrome were raised with the haloperidol injection [17,23]. In the group of patients with TBI, particularly those with additional alcohol intoxication and presenting with severe agitation and aggressive behaviour, achievement of rapid sedation can be challenging. In small series, haloperidol and olanzapine are efficacious in achieving sedation within 45 minutes. However, a blinded, randomised, controlled study with numerous patients is essential to make valid recommendations.

In the patient group with psychiatric diseases presenting to the ER with acute agitation, IM olanzapine 10 mg and haloperidol 5 mg with lorazepam 2 mg produced rapid sedation within 20 minutes in 90% and 94.1% of patients, respectively. The remaining patient in each group was sedated 25 minutes after the repeat injection, which was given 20 minutes after the first injection. The results concur with numerous reported studies on haloperidol [8,11,24] and olanzapine [7,8,11,15]. In studies using a combination of injections of haloperidol and lorazepam, similar favourable results in achieving rapid sedation have been reported [15,25]. The effectiveness of antipsychotics, by virtue of their action on various dopaminergic receptors and given the postulated mechanism of underlying agitation in various psychiatric conditions, makes them obvious and understood options.

As acute agitation in psychiatric patients is the largest studied condition and reported group, numerous other drugs have also been used to control agitation and aggressive behaviour in this group. Benzodiazepines alone or in combination with antipsychotics have been effectively used to control acute agitation in psychiatric patients in a few reported studies [26,27] and have been recommended as drugs of choice by the 2005 Scottish Intercollegiate Network Guidelines [28] and World Federation of Societies of Biological Psychiatry guidelines [29]. Although second-generation antipsychotics (in particular, olanzapine) are becoming the drugs of choice for psychiatric patients with acute agitation, haloperidol continues to be used by psychiatrists worldwide. The addition of a benzodiazepine to haloperidol was also recommended by the American Association of Emergency Psychiatry Project BETA pharmacological work group in 2012 [4] and by the Brazilian guidelines for the management of psychiatric agitation (2019) [30], primarily to reduce the incidence of extrapyramidal side effects observed with haloperidol. Most studies report a better safety profile of second-generation antipsychotics (e.g. olanzapine) compared to first-generation antipsychotics (e.g. haloperidol).

In this study, in this group of psychiatric patients with agitation, none developed any respiratory, CVS, or neurological side effects. Overall, haloperidol has been implicated in producing a higher EPS frequency than olanzapine [7,8,24,31,32]. In this study, when haloperidol was combined with a benzodiazepine (lorazepam), only one patient developed mild dystonic symptoms that resolved with anticholinergic medication.

In patients with acute agitation secondary to organic medical conditions, olanzapine has been preferred in some studies [4] and has been recommended by NICE guidelines (2019) [33] as the drug of choice. Various other studies have reported on the effectiveness of haloperidol in these cases [4,18,34]. In this study, in patients with acute agitation due to organic medical causes, IM olanzapine 10 mg was superior to IM haloperidol 5 mg in achieving rapid sedation within 20 minutes of the injection (86.4% vs 16.6%, respectively), which is statistically significant (p = 0.003). Although olanzapine was significantly superior to haloperidol in this study, more studies with numerous patients, with a good safety profile in sedating patients with acute agitation secondary to other medical conditions, are necessary before a definitive recommendation could be made.

In this study, the efficacy and safety were evident for IM olanzapine in treating Indian patients with acute undifferentiated agitation and agitation in certain etiological groups. However, the study limitations include the sample size and randomisation, necessitating further studies with more patients in each of the aetiological groups, with double-blinded randomisation, and preferably multicentrically, before any definitive recommendation can be made.

## Conclusions

IM olanzapine 10 mg is an effective and safe treatment for rapid sedation in acute undifferentiated agitation in the ER in Indian patients. In patients with acute agitation secondary to alcohol intoxication, haloperidol 5 mg is more effective than olanzapine 10 mg in achieving rapid sedation. However, the sample size was too small for a definitive recommendation. In patients with acute agitation with TBI, haloperidol and olanzapine were effective to some extent in achieving rapid sedation. Haloperidol fared marginally better at 44.4% vs 33.3% with olanzapine; however, it was statistically insignificant. In patients with psychiatric diseases presenting with acute agitation, olanzapine 10 mg and haloperidol 5 mg were equally efficacious in achieving rapid sedation (90% and 94.1%, respectively). In patients with acute agitation secondary to organic medical conditions, injection of olanzapine was superior to haloperidol in achieving rapid sedation at 86.4% and 16.6%, respectively (p = 0.003). At the studied doses, olanzapine and haloperidol were equally safe for Indian patients. The side effects of both drugs were minimal and mild. Studies with large sample sizes and double-blind randomisation comparing two or more drugs are necessary to confirm these objectives and offer definitive recommendations.
